# RNA Sequencing Analysis of the *msl2msl3, crl*, and *ggps1* Mutants Indicates that Diverse Sources of Plastid Dysfunction Do Not Alter Leaf Morphology Through a Common Signaling Pathway

**DOI:** 10.3389/fpls.2015.01148

**Published:** 2015-12-22

**Authors:** Darron R. Luesse, Margaret E. Wilson, Elizabeth S. Haswell

**Affiliations:** ^1^Department of Biological Sciences, Southern Illinois University EdwardsvilleEdwardsville, IL, USA; ^2^Department of Biology, Washington University in Saint LouisSaint Louis, MO, USA

**Keywords:** ion homeostasis, MscS-Like, RNA-seq, plastid, retrograde signaling, variegation, leaf morphology

## Abstract

Determining whether individual genes function in the same or in different pathways is an important aspect of genetic analysis. As an alternative to the construction of higher-order mutants, we used contemporary expression profiling methods to perform pathway analysis on several *Arabidopsis thaliana* mutants, including the *mscS-like* (*msl*)2*msl3* double mutant. MSL2 and MSL3 are implicated in plastid ion homeostasis, and *msl2msl3* double mutants exhibit leaves with a lobed periphery, a rumpled surface, and disturbed mesophyll cell organization. Similar developmental phenotypes are also observed in other mutants with defects in a range of other chloroplast or mitochondrial functions, including biogenesis, gene expression, and metabolism. We wished to test the hypothesis that the common leaf morphology phenotypes of these mutants are the result of a characteristic nuclear expression pattern that is generated in response to organelle dysfunction. RNA-Sequencing was performed on aerial tissue of *msl2msl3* *geranylgeranyl diphosphate synthase 1* (*ggps1*), and *crumpled leaf* (*crl*) mutants. While large groups of co-expressed genes were identified in pairwise comparisons between genotypes, we were only able to identify a small set of genes that showed similar expression profiles in all three genotypes. Subsequent comparison to the previously published gene expression profiles of two other mutants, *yellow variegated 2* (*var2*) and *scabra3* (*sca3*), failed to reveal a common pattern of gene expression associated with superficially similar leaf morphology defects. Nor did we observe overlap between genes differentially expressed in *msl2msl3, crl*, and *ggps1* and a previously identified retrograde core response module. These data suggest that a common retrograde signaling pathway initiated by organelle dysfunction either does not exist in these mutants or cannot be identified through transcriptomic methods. Instead, the leaf developmental defects observed in these mutants may be achieved through a number of independent pathways.

## Introduction

Ion homeostasis across organelle membranes is critical for plant survival. Proper solute concentrations in the stroma are essential for organelle size and shape, and the ions themselves are required for a wide variety of metabolic processes. The establishment and maintenance of ion homeostasis in the plant plastid is therefore a critical yet complex biological process. The genetic disruption of Fe^2+∕3+^ (Duy et al., 2007; Jeong et al., 2008), Na^+^ (Müller et al., 2014), or K^+^ (Kunz et al., 2014) homeostasis in the plastid can lead to impaired chloroplast function, reduced photosynthetic capacity, and stunted growth.

Two proteins likely to be involved in ion homeostasis in plastids are MscS-Like (MSL)2 and MSL3, two Arabidopsis homologs of MscS, a well-studied mechanosensitive ion channel from *Escherichia coli* (Hamilton et al., 2015a). Several lines of evidence support the proposal that MSL2 and MSL3 function as mechanically gated ion channels. At least two members of the Arabidopsis MSL family have been shown to function as mechanically-gated ion channels by single-channel patch clamp electrophysiology (Maksaev and Haswell, 2012; Hamilton et al., 2015b), and MSL3 is capable of rescuing an *E. coli* strain that lacks key mechanosensitive channels (Haswell and Meyerowitz, 2006).

MSL2 and MSL3 localize to the plastid envelope, and are semi-redundantly required for normal plastid size and shape, plastid division, and plastid osmoregulation (Haswell and Meyerowitz, 2006; Wilson et al., 2011; Veley et al., 2012). The large, round phenotype of epidermal leaf plastids in the *msl2msl3* mutant can be suppressed by a variety of genetic, physiological, and media manipulations that result in increased cytoplasmic osmolarity, indicating that the primary defect in this mutant is an inability to release osmolytes, most likely ions, from inside the plastid during hypoosmotic stress (Veley et al., 2012).

*msl2msl3* mutants also exhibit a variety of leaf architecture phenotypes. Most prominently, they exhibit white or light green patches on the leaf surface, corresponding to regions of leaf tissue with large air spaces between mesophyll cells (Haswell and Meyerowitz, 2006). This indicates a tissue- as well as a cell-level defect, and in this respect, *msl2msl3* mutants resemble variegation mutants. The classic variegation mutants—some of the first mutants ever to be isolated in Arabidopsis—are derived from defects in chloroplast biogenesis and include *chloroplast mutator (chm), yellow variegated (var)1, yellow variegated (var)2*, and *immutans (im)* (Foudree et al., 2012). The *var2* mutant phenotype is caused by a mutation in a subunit of the thylakoid-localized FtsH complex, which is likely involved in chloroplast biogenesis and development (Chen et al., 2000; Sakamoto et al., 2003). Complete loss of the FtsH complex leads to arrest of chloroplast development, while slight disruptions result in variegated plants (Liu et al., 2010). *var2* mutants show variegation phenotypes of varying intensity. While most have at least small white patches around the leaf periphery, more severely affected leaves are smaller than wild-type and have larger, irregularly-shaped white segments throughout the leaf, as well as scalloping at the leaf margin (Liu et al., 2010).

In addition to patches of white on the leaves, *msl2msl3* mutants are also dwarfed and have rumpled leaves and uneven leaf margins (Jensen and Haswell, 2012; Wilson et al., 2014). A straightforward explanation for these data is that MSL2 and MSL3 form MS ion channels in the plastid envelope, and their absence leads to defects in plastid osmoregulation and ion homeostasis, which in turn leads to pleiotropic effects on leaf development. We note that a defect in plastid ion homeostasis in the *msl2msl3* mutant has not yet been directly shown, and it is currently unclear how disruptions in plastid ion homeostasis might impinge upon pathways that regulate leaf development.

It has previously been proposed that leaf development is sensitive to organelle function (Streatfield et al., 1999; Tan et al., 2008; Moschopoulos et al., 2012), possibly through the action of an organelle-to-nucleus, or retrograde, signaling pathway (recent reviews on this topic include Barajas-López Jde et al., 2013; Jarvis and López-Juez, 2013). In favor of this argument, a large number of mutants with lesions in nuclear genes that encode plastid- or mitochondrial-targeted proteins exhibit similar leaf phenotypes (summarized in Moschopoulos et al., 2012). Patchy leaf color and leaf morphology defects are observed in plants harboring mutations in chloroplast division (Asano et al., 2004), chlorophyll synthesis (Ruppel et al., 2013), double strand break repair (Maréchal et al., 2009; Lepage et al., 2013), ribosomal RNA synthesis and processing (Bellaoui and Gruissem, 2004; Hricová et al., 2006), organellar tRNA synthesis (Uwer et al., 1998; Moschopoulos et al., 2012), and mitochondrial protease activity (Gibala et al., 2009). While these leaf morphology defects show no obvious link to the primary function of their causal mutation, all of these lesions are likely to produce physiological conditions that lead to organelle dysfunction. One possible explanation for these results is an organelle-to-nucleus signaling pathway initiated by a variety of plastid dysfunctions, which then produces a characteristic nuclear gene expression pattern that leads to defective leaf morphology.

Like the *msl2msl3* mutant, the *crumpled leaf* (*crl*) mutant also exhibits overall small stature and white sectors on its leaves, which have a ruffled surface and irregular margins (Asano et al., 2004). *CRL* encodes a novel protein of the chloroplast outer envelope and *crl* mutants have a defect in plastid replication so severe that some leaf cells in the mature plant lack any plastids (Chen et al., 2009). While chlorophyll a and b content is decreased in the *crl* mutant—likely a result of fewer plastids—photochemistry and thylakoid organization are indistinguishable from wild type plants, suggesting normal chloroplast biogenesis (Asano et al., 2004). Spontaneous microlesion formation and enhanced resistance to bacterial infection have been documented in *crl* mutants (Šimková et al., 2012). Cell cycle regulation and root meristem cell differentiation are also disrupted (Hudik et al., 2014).

A subset of the leaf morphology phenotypes observed in the *msl2msl3, var2*, and *crl* mutants are displayed by the *ggps1-1* mutant, which harbors a temperature-sensitive allele of *GERANYLGERANYL DIPHOSPHATE SYNTHASE (GGPS)1. GGPS1* is an essential gene that encodes a key branch point enzyme required for plant isoprenoid biosynthesis (Bouvier et al., 2005). The GGPS1 protein is localized to the plastid (Okada et al., 2000). T-DNA insertions that disrupt the DNA sequence encoding the chloroplast targeting peptide and the C-terminus of the GGPS1 protein lead to seedling albino and embryo lethal phenotypes, respectively, while the EMS-induced single amino acid change in the *ggps1-1* allele was shown to produce temperature-sensitive variegation with reproducible patterning (Ruppel et al., 2013). Mutant plants grown at 21–23°C have an albino region in the center of the leaf and green tissue at the periphery, and have leaves that are typically smaller than wild type with a slightly rumpled surface and scalloped edges. Growth at higher temperatures leads to an increase in both the size of albino region and the severity of scalloping at the leaf margin. Transmission electron microscopy of white sectors revealed plastids with poorly developed thylakoid membranes.

A number of other Arabidopsis mutants show variable leaf color, rumpled leaves, and serrated or scalloped leaf edges; all harbor lesions in genes that encode plastid- or mitochondria-targeted proteins. These proteins include CAB UNDEREXPRESSION (CUE)1, a phosphoenolpyruvate (PEP)/phosphate translocator of the plastid inner envelope membrane (Li et al., 1995; Streatfield et al., 1999; Staehr et al., 2014); WHIRLY1/WHIRLY2, which is involved in double strand break repair and plastid genome stability (Maréchal et al., 2009; Lepage et al., 2013); DEFECTIVE CHLOROPLASTS AND LEAVES (DCL), which is involved in plastid ribosomal RNA processing in Arabidopsis (Bellaoui and Gruissem, 2004); SCABRA3, a plastid-localized RNA polymerase (Hricová et al., 2006); EMBRYO DEFECTIVE DEVELOPMENT (EDD1), an organellar glycyl-tRNA synthetase (Uwer et al., 1998; Moschopoulos et al., 2012); and the mitochondrial protease AtFtsH4 (Gibala et al., 2009). Similar mutants have been isolated in *Antirrhinum*, tobacco, and tomato (Chatterjee et al., 1996; Keddie et al., 1996; Wang et al., 2000; Wycliffe et al., 2005).

Taken together, these data suggest that the proper function of endosymbiotic organelles is required for normal leaf patterning. Because lesions in such a large number of genes implicated in apparently unrelated plastid or mitochondrial processes result in similar defects in leaf morphology, it is unlikely that these phenotypes are a direct result of the disruption of a specific metabolic or biosynthetic pathway within an organelle. Thus, while the primary defects are only similar in that they are defects in plastid function—they disrupt different pathways and likely generate variegated leaves through different mechanisms—all the mutants take on a similar appearance in the development of the leaf surface and edges. One possible explanation is that a characteristic nuclear gene expression pattern may be generated in response to a variety of organellar dysfunctions, and that this expression pattern leads to defects in leaf development and morphology. A second possible explanation is that the defects in leaf development and morphology seen in these mutants may be produced by different pathways that only coincidently produce similar developmental phenotypes that are not related at the molecular level.

One way to test for the presence of a gene pathway leading from plastid division to leaf development would be to make higher-order mutants, combining lesions in in the same background to look for genetic interactions (Huang and Sternberg, 1995; Koornneef et al., 2006). Instead, we characterized the gene expression profiles of *msl2msl3, crl*, and *ggps1* mutant leaf tissue by RNA sequencing, and compared the results to previously published expression profiles of two other mutants, *var2* and *sca3* and to 39 genes previously identified as a possible retrograde core response module (Glaßer et al., 2014). We reasoned that a high degree of overlap between the transcript profiles of these mutants would lend support to the proposal that lesions affecting different aspects of organelle physiology result in similar whole-cell and plant phenotypes because they alter gene expression networks in the same way. On the other hand, little overlap between the gene expression profiles of the mutants would suggest that diverse gene expression networks can still produce highly similar leaf phenotypes. It was expected that either of these outcomes would help illuminate the genetic pathway or pathways that link defects in plastid function in general (and in plastid ion homeostasis in particular) to disruptions in leaf development.

## Materials and methods

### Plant growth

Seeds were sown on moist potting mix and stratified at 4°C for 48 h. Plants were grown in a growth chamber under 16 h photoperiods with a light intensity of 100–120 μmol m^−2^ s^−1^ at 21°C. The *ggps1-1* and *msl2-3* (GK-195D11) alleles are in the Columbia (Col-*0*) background. The *msl3-1* allele is in the Wassilewskija (Ws) background (Haswell and Meyerowitz, 2006). The *crl-3* mutant line (GK_714E08) was identified in the GABI-Kat collection of T-DNA insertion lines (Rosso et al., 2003) and is in the Col-*0* background.

### Microscopy

Confocal laser scanning microscopy was performed on the youngest leaves of 3-week-old soil-grown plants using a FLUOVIEW FV1000 (Olympus), and images were captured with FVIO-ASW software. Chlorophyll autofluorescence was excited at 635 nm and emissions collected with a 655–755 nm band-pass filter.

### Semi-quantitative reverse transcription PCR

RNA was isolated from pools of 2-week-old Col-*0* and *crl-3* seedlings using the RNeasy Plant Mini kit (Qiagen) with an on-column DNase digestion. One milligram of RNA was used for subsequent cDNA synthesis with an oligo dT primer. The following primers were used to amplify *CRL* transcript levels: (a) 5′-ATGGGTACCGAGTCGGGTT-3′, (b) 5′-GGAGCAAGCGTCAAGAGATCCTG-3′, and (c) 5′-CCAGGTGAAGTTGAGCCTTCGTAC-3′. The *ACTIN* gene was used as a loading control and was amplified with the primers ACT.F2 5′-TACGCCAGTGGTCGTACAAC-3′ and Actin.8 5′-AACGACCTTAATCTTCATGCTGC-3′.

### Tissue collection, RNA isolation, and RNA sequencing

Aerial tissue from 3-week-old *msl2msl3* and *crl* mutants was harvested in triplicate, along with co-grown wild-type Col-*0* controls. This time point was chosen to collect the largest rosettes possible prior to bolting. Newer leaves near the center of the rosette were collected from 5-week-old *ggps1-1* mutants and controls in triplicate. These plants were co-cultivated with the *msl2msl3* and *crl* mutants, but were harvested later to allow leaves to reach sufficient size for dissection. After harvesting, leaves were dissected with a razor blade to isolate white tissue for sequencing.

RNA was isolated from three biological replicates per mutant using the RNeasy Plant Mini kit (Qiagen) with an on-column DNase digestion. Each sample had a 260:280 ratio of at least 2.0 and a RIN value above 8.0. Library preparation and RNA-seq was performed at the Genome Technology Access Center at Washington University. Samples were enriched for mRNA using a Dyna mRNA Direct kit (Life Technologies). One biological replicate from each mutant and its corresponding wild-type control were barcoded and run together using paired-end reads on an Illumina Hi-seq 2500. This was performed in triplicate, resulting in 15 individual sequenced samples.

### Analysis of RNA-sequencing reads

Individual reads from each sample were aligned to the TAIR10 Arabidopsis reference genome provided by http://support.illumina.com/sequencing/sequencing_software/igenome.html using STAR (Dobin et al., 2013). A list of differentially expressed genes was determined for each sample in edgeR by comparison to the appropriate wild-type control (Robinson et al., 2010). Significant expression changes in *msl2msl3* and *crl* backgrounds were determined by comparison to the 3-week-old wild type controls grown and harvested with these mutants. For *ggps1*, significant expression changes were determined by comparison with 5-week-old wild-type leaf tissue that was grown and harvested with the mutant. Different wild-type standards were used to eliminate genes from consideration that showed altered expression based on age or tissue type. Lists were filtered to omit genes with a false discovery rate (FDR) below 0.05. A log_2_ transformation was performed on all fold-change data.

### Analysis of RNA-sequencing results

The hypergeometric distribution test was used to assess the likelihood that any overlap between the differentially expressed genes in two mutants is nonrandom. The hypergeometric *P*-value is calculated using the equation *p* = *(*_*k*_*C*_*x*_*) (*_(*n*−*k*)_*C*_(*n*−*x*)_*)/*_*N*_*C*_*n*_, where *N* is the number of genes in the genome, *k* is the number of genes identified in the first sample, *n* is the number of genes identified in the second sample, *x* is the number of overlapping genes and _*k*_*C*_*x*_ is the number of possible gene combinations.

The online tool g:Profiler was used to search for significantly over-represented GO terms within the gene lists (Reimand et al., 2007). *Arabidopsis thaliana* was set as the reference organism. The default g:SCS algorithm was used to determine the significance threshold and correct for the problem of multiple testing. For these analyses, a transformed *p*-value of 0.05 was established as the cutoff for significantly represented GO terms.

### Comparison with previously reported microarray results

Microarray results for the *sca3* mutant were previously published (Hricová et al., 2006). While raw data were not available, supplementary information included a list of all genes from the original analysis with an absolute fold change greater than ±1.5-fold and a *p*-value less than 0.05. Microarray data from *var2* mutant seedlings (Miura et al., 2010) were obtained from NCBI's Gene Expression Omnibus (GEO Accession GSE18646) and analyzed using the GEO2R tool. Gene expression from white sectors of *var2* leaves was compared to leaves from the control wild-type Col-*0*, also available from GEO as part of the original Miura et al data set. A list of potential candidate genes that may be involved in retrograde signaling in response to alterations of chloroplast performance was obtained from Glaßer et al. (2014).

## Results

### RNA-sequencing data collection

**Figure 1:**
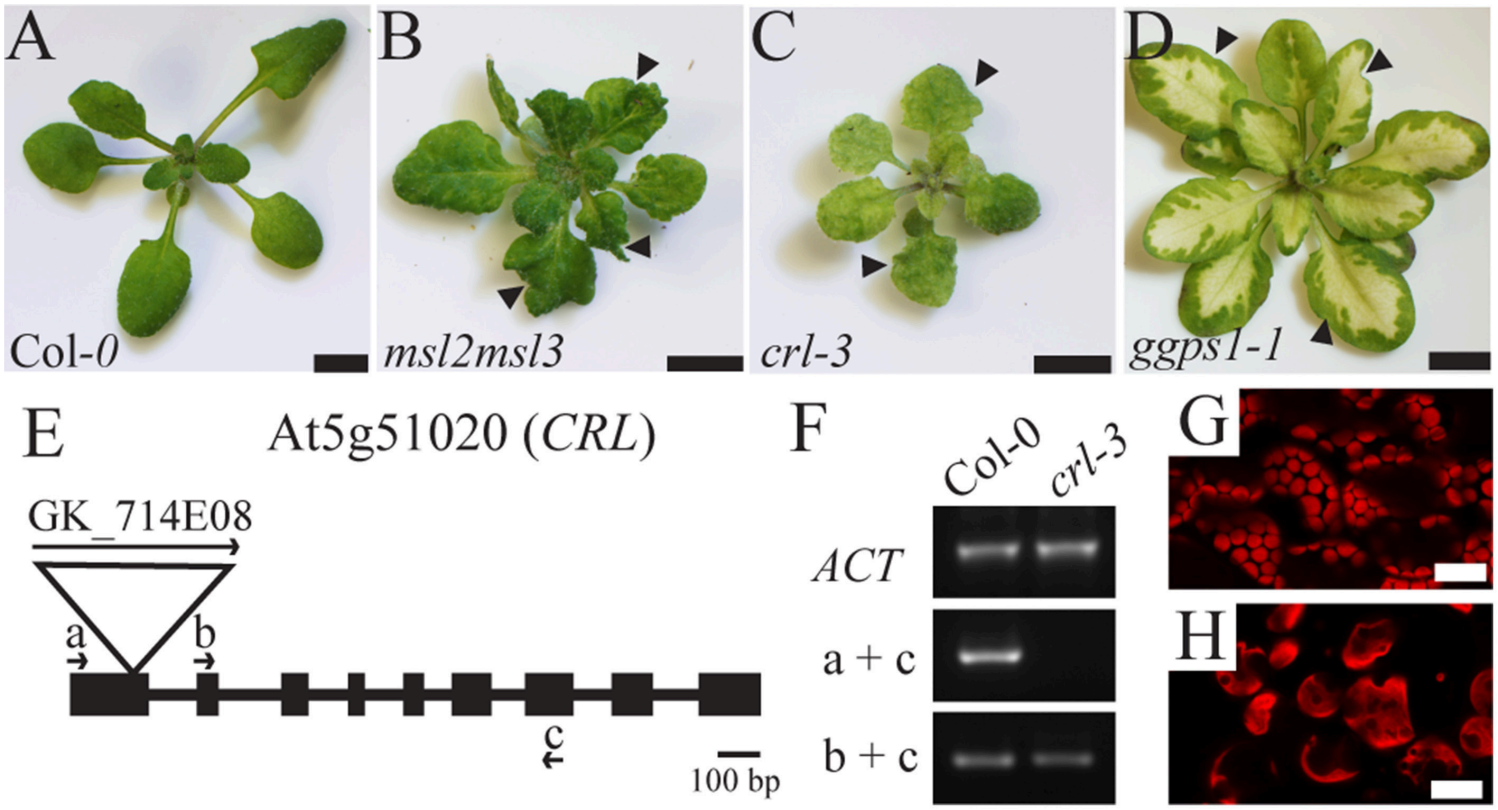
**Aerial phenotypes of ***msl2msl3***, ***crl***, and ***ggps1*** mutants and characterization of ***crl-3*** allele**. Images of 3-week-old Col-*0* **(A)**, *msl2msl3* **(B)**, *crl* **(C)**, and 5-week-old *ggps1* **(D)** mutants. Arrows in **(B–D)** indicate notched leaf margins. Scale bar = 5 mm. Schematic of the *CRL* gene. The T-DNA insertion in *crl-3* is indicated by a triangle. According to GABI-KAT, the insertion site is 134 bp downstream of the *CRL* start codon, near the end of exon 1 **(E)**. Boxes and lines indicate exons and introns, respectively. Arrows indicate the location of primers used in **(F)**. RT-PCR on cDNA derived from Col-*0* and *crl-3* seedlings **(F)**. No *CRL* transcript was detected using primer pairs spanning the T-DNA insertion site in the *crl-3* mutant background. Confocal micrographs of mesophyll chloroplasts of Col-*0* **(G)** and *crl-3* **(H)** rosette leaves. Scale bar = 20 μm.

Three mutants with diverse plastid defects—in osmoregulation and likely ion homeostasis (*msl2msl3*, Figure 1B), in plastid division and partitioning (*crl* Figure 1C), and in chlorophyll biosynthesis (*ggps1*, Figure 1D)—all display patchy, rumpled leaves with notched margins (arrows in Figures 1B–D). While the primary defect and the likely mechanisms by which variegation is created are different in the mutants selected for analysis, the secondary leaf morphology defects are at least superficially similar. To determine if these secondary leaf morphology defects were more than superficially similar, and were instead generated by a common signaling pathway triggered by plastid dysfunction, RNA-seq analysis was performed on leaf tissue from *msl2-3 msl3-1* double mutants (referred to as *msl2msl3* hereafter), *crl-3* single mutants (referred to as *crl* hereafter), and albino leaf tissue from *ggps1-1* (referred to as *ggps1* hereafter). The *msl2-3* allele is a T-DNA insertion in the first exon of *MSL2* (At5g10490, (Wilson et al., 2011) and the *msl3-1* allele is a T-DNA insertion in the last exon of *MSL3* (At1g58200, Haswell and Meyerowitz, 2006). A previously undescribed *CRL* mutant allele(At5g51020), named here *crl-3*, is a T-DNA insertion in the first exon of the gene. *CRL* transcript levels downstream of the T-DNA insertion site were similar to those observed in wild-type plants; however, no transcript spanning the T-DNA insertion site was detected (Figures 1E,F). These data suggest that *crl-3* mutant plants are unable to produce a functional full-length protein, either as a result of the incorporation of T-DNA sequences into the coding portion of the mRNA and/or truncation of wild type N-terminal sequences. The *crl-3* mutant is small, with crumpled leaves and enlarged chloroplasts (Figures 1C,H), as shown for other *crl* mutant alleles (Asano et al., 2004; Šimková et al., 2012). The *ggps1-1* allele contains a missense point mutation in a conserved aspartic acid of GGPS1 (encoded by At4g36810), resulting in a temperature-sensitive phenotype (Ruppel et al., 2013).

RNA was collected from three biological replicates of each mutant and co-cultivated wild-type control plants. Between 43 and 63 million read pairs were mapped to the Arabidopsis reference genome for each genotype and its appropriate control using STAR (Dobin et al., 2013). For each genotype, this represented at least 94% of the total reads uniquely mapping, greater than 21,000 genes identified, and at least 13,000 genes with five counts per million per replicate. The ribosomal fraction was less than 0.25%. Gene-level transcript abundance and differential expression was determined by edgeR (Robinson et al., 2010).

Significant expression changes in *msl2msl3* and *crl* backgrounds were determined by comparison to the 3-week-old wild type controls grown and harvested with these mutants. In the *msl2msl3* mutants 4777 genes with an FDR less than 0.05 were identified. The *crl* mutant showed differential expression of 3499 genes. For *ggps1*, significant expression changes were determined by comparison with 5-week-old wild-type leaf tissue that was grown and harvested with the mutant. Different wild-type standards were used for *ggps1* to enhance downstream comparisons with *msl2msl3* and *crl*, eliminating genes from consideration that showed altered regulation based on age or tissue type. In the *ggps1-1* white tissue, 8121 genes with an FDR less than 0.05 were identified. The larger number of differentially regulated genes discovered in *ggps1* when compared to the other mutants may be a result of the greater severity of the albino phenotype (Figure 1D).

**Figure 2:**
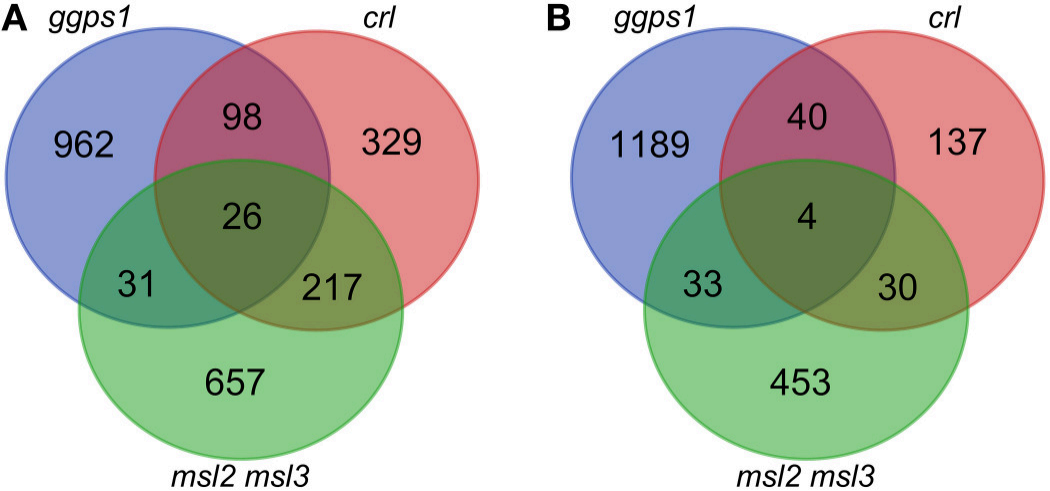
**Overlap of differentially expressed genes in ***msl2msl3, crl***, and ***ggps1*** mutants**. Lists of up-regulated genes **(A)** or down-regulated genes **(B)** were compared for the three mutants. Venn diagrams include all genes with an FDR below 0.05 and an absolute LFC greater than 1.65.

In agreement with a role for MSL2 and MSL3 in maintaining plastid ion homeostasis, 148 out of the 2247 (6.6%) differentially regulated genes exhibiting a log_2_ fold change (LFC) of 1.17 (2.25-fold) or more were associated with the GO terms “transition metal ion transport” and “ion transport” (*p* < 0.01; marked in green in Supplementary Data Sheet 1). For further analysis, an LFC of ±1.65 was implemented as a cutoff. We chose this LFC cut-off because it corresponds to a fold change of approximately 3.1, a level of differential expression that is likely to be physiologically relevant. This resulted in 931 up-regulated and 520 down-regulated genes in the *msl2msl3* double mutant, 670 up-regulated and 211 down-regulated genes in *crl*, and 1117 up-regulated and 1266 down-regulated genes in *ggps1-1* white tissue, all compared to their respective wild type (Figures 2A,B; these gene lists are provided in Supplementary Data Sheet 1).

### Our selected mutants show distinct transcriptomic profiles

Differentially expressed genes from each mutant were compared and a hypergeometric test performed for genes identified in each sample in a pair-wise fashion to reveal potential relationships between transcriptomic profiles of individual mutants (Table 1). The hypergeometric distribution test is a statistical measure of the probability that the number of genes differentially expressed in two different mutants could arise by chance, given the number of possible genes. Most pairwise comparisons had a hypergeometric distribution *p*-value of less than 0.01, suggesting that the similarities in the gene expression patterns of *msl2msl3* and *crl* (or *crl* and *ggps1*) are unlikely to have occurred randomly. The one exception was comparison between *msl2msl3* and *ggps1*. These mutants showed very little overlap in either up-regulated or down-regulated genes and both hypergeometric distribution *p*-values were greater than 0.01. This suggests that *msl2msl3* and *ggps1* respond to plastid dysfunction with distinct genetic responses, while the other pairs of mutants may share pathways or pathway components.

**Table 1:** **Pairwise hypergeometric probability test shows a statistically significant overlap in differentially expressed genes between ***msl2msl3*** and ***crl*** and between ***crl*** and ***ggps1*****.

**Change compared to the wild type**	**Mutant 1 (Number of genes)**	**Mutant 2 (Number of genes)**	**Number of genes altered in both Mutant 1 and Mutant 2**	**Hypergeometric probability**
Up-regulated	*msl2msl3* (931)	*crl* (670)	243	4.48 e^−55^
Up-regulated	*msl2msl3* (931)	*ggps1* (1117)	57	0.95
Up-regulated	*crl* (670)	*ggps1* (1117)	124	6.88 e^−44^
Down-regulated	*msl2msl3* (520)	*crl* (211)	34	1.27 e^−20^
Down-regulated	*msl2msl3* (520)	*ggps1*(1266)	37	0.021
Down-regulated	*crl* (211)	*ggps1* (1266)	44	2.99 e^−16^

*Probability of the observed overlap based on size of the differentially expressed gene list for each (in parentheses) was calculated. Gray shading indicates significance with a cutoff of 0.01*.

We next attempted to identify a common set of differentially expressed genes present in all samples. Twenty-six genes were identified as up-regulated in leaf tissue from *msl2msl3* double mutants, *crl-3* mutants, and dissected white tissue from *ggps1-1* seedlings (Table 2 and Figure 2A). Surprisingly, none of the protein products from these genes are predicted to localize to plastids (Ashburner et al., 2000). Six of these proteins are predicted to reside within mitochondria and five to the plasma membrane. Two of the plasma membrane proteins, At3g28600 and At5g45570, are predicted to function as hydrolases, and two others are potential receptors, an S-locus receptor kinase that may bind mannose and a Leucine Rich Repeat receptor-like protein. At3g16450 is another potential sugar sensor and a predicted nuclear protein. Five proteins from this group are also predicted to reside within the cell wall, and may play a role in cell expansion. Finally, seven of the 26 proteins in this set are predicted to localize to the nucleus and alter transcription.

**Table 2:** **Twenty-six genes were up-regulated in ***msl2msl3, crl***, and ***ggps1*****.

**Gene**	**Description**	***msl2msl3***		***crl***		***ggps1***	
		**LFC**	**FDR**	**LFC**	**crl FDR**	**LFC**	**FDR**
AT1G19610^*^	Pathogen-related defensin-like protein	2.49	5.1 e^−08^	3.71	3.3 e^−16^	3.23	9.1 e^−21^
AT2G02990	*RNS1*, Ribonuclease 1	2.37	8.6 e^−07^	4.20	1.2 e^−20^	1.86	1.7 e^−03^
AT5G14180	*MPL1, Myzus persicae*-induced lipase 1 involved in lipid metabolism	2.11	1.8 e^−04^	3.42	3.5 e^−11^	5.89	6.1 e^−30^
AT5G40990^*^	*GLIP1*, GDSL lipase 1	4.04	1.4 e^−03^	5.28	8.1 e^−06^	5.48	4.2 e^−03^
AT3G20370	TRAF-like family protein	2.12	7.8 e^−07^	1.83	3.4 e^−05^	4.41	3.2 e^−03^
AT1G21310^*^	*ATEXT3*, Extensin 3	2.22	4.6 e^−21^	3.13	2.2 e^−38^	2.06	2.1 e^−05^
AT3G01600^*^	*ANAC044*, NAC domain containing DNA binding protein 44	3.91	7.7 e^−32^	2.94	3.1 e^−17^	2.62	1.7 e^−02^
AT5G61890	Encodes a member of the ERF (ethylene response factor) subfamily B-4 of ERF/AP2 transcription factor family	3.54	1.2 e^−07^	2.43	3.2 e^−03^	3.35	2.6 e^−06^
AT5G18270^*^	*ANAC087*, NAC domain containing DNA binding protein 87	3.07	1.1 e^−34^	2.91	2.3 e^−31^	1.70	2.0 e^−05^
AT5G46590	*ANAC096*, NAC domain containing DNA binding protein 96	2.77	5.8 e^−08^	1.90	1.0 e^−03^	1.72	4.9 e^−02^
AT2G38250^*^	Homeodomain-like superfamily DNA binding protein	2.54	1.4 e^−14^	2.38	1.4 e^−12^	2.45	6.5 e^−08^
AT5G13330	*Rap2.6L*, Encodes a member of the ERF (ethylene response factor) subfamily B-4 of ERF/AP2 transcription factor family	2.51	4.7 e^−15^	1.95	8.9 e^−09^	4.04	1.7 e^−18^
AT3G16450	Mannose-binding lectin superfamily protein	2.22	1.6 e^−07^	2.74	7.1 e^−11^	2.61	3.7 e^−02^
AT3G06490	*MYB108*, Putative transcription factor myb domain protein 108	1.70	3.9 e^−02^	1.76	4.2 e^−02^	1.89	3.4 e^−06^
AT4G29200	Beta-galactosidase related protein	9.54	7.9 e^−34^	9.10	9.6 e^−28^	5.30	5.6 e^−03^
AT4G04030	*ATOFP9*, Ovate family protein 9	6.05	1.3 e^−04^	5.16	6.0 e^−03^	5.21	6.2 e^−03^
AT5G15360	unknown protein	5.93	9.1 e^−12^	4.83	1.3 e^−06^	7.34	8.6 e^−01^
AT5G03090	BEST *Arabidopsis thaliana* protein match is: mto 1 responding down 1	4.90	1.5 e^−06^	3.64	6.6 e^−03^	8.03	3.8 e^−15^
AT3G01345	Expressed protein. InterPro DOMAIN/s: Glycoside hydrolase, family 35 (InterPro:IPR001944)	3.45	2.2 e^−07^	1.99	1.9 e^−02^	4.07	1.5 e^−07^
AT1G65500^#^	Unknown protein	1.74	9.0 e^−11^	2.14	1.2 e^−15^	1.78	5.5 e^−05^
AT5G45570	Ulp1 protease family protein	4.66	2.5 e^−02^	4.56	4.4 e^−02^	4.20	3.9 e^−04^
AT2G32660^*^	*ATRLP22*, Receptor like protein 22 with Leucine Rich Repeat	3.52	1.8 e^−02^	3.83	1.2 e^−02^	1.79	4.9 e^−03^
AT1G61440	S-locus lectin protein kinase family protein	5.40	1.5 e^−28^	1.72	4.4 e^−02^	4.52	3.5 e^−02^
AT3G28600	P-loop containing nucleoside triphosphate hydrolases superfamily protein	4.27	9.0 e^−04^	3.46	1.9 e^−02^	4.66	1.9 e^−06^
AT3G02810	Protein kinase superfamily protein	4.23	2.5 e^−10^	4.74	3.1 e^−13^	1.84	3.4 e^−02^
AT4G08093	Pseudogene of unknown protein	7.18	8.7 e^−46^	3.24	1.7 e^−06^	2.75	4.3 e^−04^

# *Indicates gene is also up-regulated in sca3*.

* *Indicates gene is also up-regulated in var2*. *Color indicates predicted subcellular localization: blue, cell wall; yellow, nucleus; red, mitochondria; and orange, plasma membrane. No color indicates no prediction*.

Venn analysis also revealed four genes that were down-regulated in all three mutants (Table 3 and Figure 2B). The product of one of these, PUMILLO9, is localized to the cytoplasm and involved in mRNA stability (Francischini and Quaggio, 2009). Two others potentially have enzymatic activity; At3g45940 encodes a glycosyl hydrolyase that is found in the apoplast and At1g12010 encodes a cytoplasmic oxidase (Ashburner et al., 2000).

**Table 3:** **Four genes were down-regulated in ***msl2msl3, crl***, and ***ggps1*****.

**Gene**	**Description**	***msl2msl3***		***Crl***		***ggps1***	
		**LFC**	**FDR**	**LFC**	**FDR**	**LFC**	**FDR**
AT3G45940	*XYL2*, Glycosyl hydrolases family 31 protein	−2.97	5.1 e^−09^	−3.25	3.72 e^−04^	−2.28	2.5 e^−03^
AT1G67260	*TCP1*, TCP family transcription factor	−6.83	9.8 e^−04^	−3.16	9.6 e^−07^	−3.21	1.1 e^−08^
AT1G35730	*APUM9*, Encodes a member of the Arabidopsis Pumilio (APUM) proteins containing PUF domain. PUF proteins regulate both mRNA stability and translation through sequence-specific binding to the 3′ UTR of target mRNA transcripts	−3.23	2.1 e^−05^	−2.99	4.4 e^−04^	−3.10	1.3 e^−04^
AT1G12010	2-oxoglutarate (2OG) and Fe(II)-dependent oxygenase superfamily protein	−2.99	1.5 e^−17^	−3.19	3.3 e^−11^	−7.67	1.2 e^−25^

*Shading indicates predicted subcellular localization: blue, cell wall; yellow, nucleus; brown, cytoplasm*.

### The overlapping gene list for all three mutants does not, though some pairwise lists do, contain statistically over-represented go terms

While the 30 genes differentially expressed in *msl2msl3, crl*, and *ggps1* mutants are candidates for a shared response pathway, their differential expression does not guarantee that they are involved in the manifestation of the rumpled leaf phenotype. To determine if the two sets of overlapping differentially expressed genes share specific biological pathways or functions, the Gene Ontology (GO) terms associated with each were analyzed with g:Profiler (Reimand et al., 2007). This tool analyzes the GO terms associated with each gene in a list and determines if any specific biological processes, cellular locations, or molecular functions are represented in the list beyond what is expected by chance. Analysis of both the up-regulated and the down-regulated genes did not reveal significant enrichment for any specific GO terms. This suggests that these genes are unlikely to work in a coordinated manner. However, it is also possible that the genes are working together in an undescribed pathway that is not reflected in the GO terms.

We also performed GO-term analysis of differentially regulated genes found in just two genotypes (Supplementary Data Sheet 2). Lists of differentially regulated genes found in both *ggps1* and *crl* showed significant (*p* < 0.05) representation of several GO terms indicating secondary metabolic processes, immune and defense response, peptide-methionine (R)-S-oxide reductase activity, and responses to external and biotic stimuli, other organisms, stress and iron starvation. Analysis of genes differentially regulated in both *msl2msl3* and *crl* showed significant (*p* ≤ 0.05) over-representation of genes involved in S-glycoside catabolic process, nitrile biosynthetic process, and biotin transport and metabolism. There were no significantly over-represented GO terms in the overlapping genes from *msl2msl3* and *ggps1*.

### Including gene expression data from *var2* and *sca3* does not reveal a core set of differentially expressed genes

If the set of genes that are differentially expressed in the *msl2msl3, crl*, and *ggps1* mutants represent a core set of genes responsible for signaling in response to plastid dysfunction to produce the notched-leaf phenotype, it would be expected that other mutants with wavy leaf margins would also show altered expression of these genes. To test this, we compared our RNA-seq data for *msl2msl3, crl*, and *ggps1* (with LFC ≥ 1.65 and FDR < 0.05) to previously published microarray expression data comparing *sca3* to wild type (Hricová et al., 2006) and *var2* to wild type (Miura et al., 2010). The differentially expressed gene list for *var2* was generated by comparing raw microarray data from *var2* white leaf tissue to wild-type leaf tissue (both from Miura et al., 2010), and filtering for LFC ≥ 1.65 and an adjusted *p* < 0.05. The differentially expressed gene list for *sca3* leaf tissue was obtained from the supplemental information in Hricová et al. (2006) which contained a list of differentially expressed genes with a fold-change of 1.5 or higher, and *p* < 0.05.

**Figure 3:**
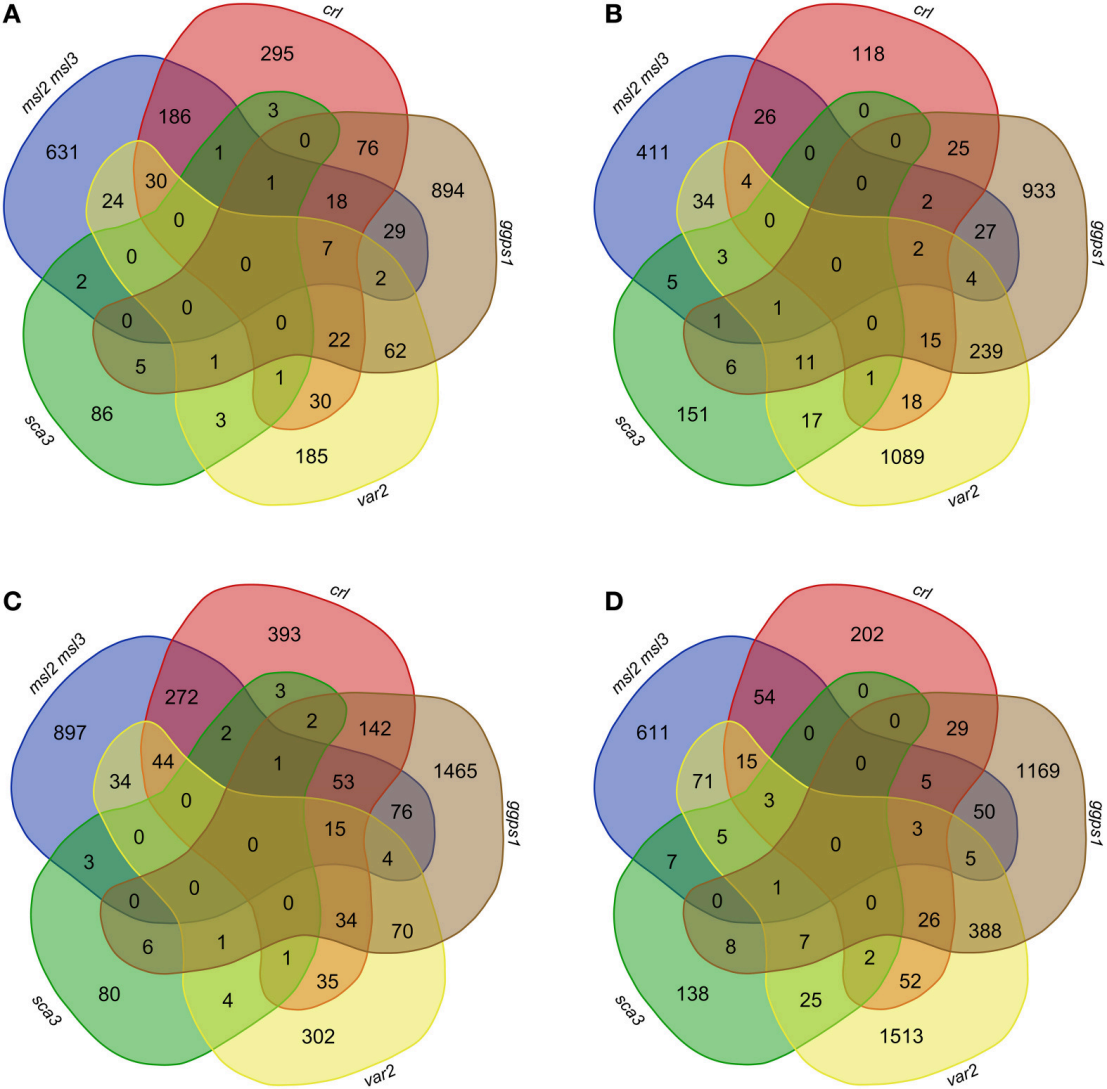
**Overlap of differentially expressed genes in ***msl2msl3, crl, ggps1, sca3***, and ***var2*** mutants**. Gene lists for each mutant were filtered by significance, removing anything with an FDR > 0.05. Up-regulated genes with an LFC ≥ 1.65 **(A)** and 1.17 **(C)** were compared in *msl2msl3, crl, ggps1, sca3*, and *var2*. Comparisons were also made with genes down-regulated with an absolute LFC ≥−1.65 **(B)** and −1.17 **(D)**. No genes were found to overlap in all samples.

Lists of up-regulated or down-regulated genes from *var2* and *sca3* were compared with those from *msl2msl3, crl*, and *ggps1*. When we used the LFC 1.65 cutoff for all but *sca3* (for which the only data available was a cutoff of 1.5 or greater), there were no genes over-expressed or under-expressed in all five mutants (Figures 3A,C). In fact, most of the genes differentially expressed in *sca3* (83.5% of up-regulated, and 77% of down-regulated genes) do not overlap with any other mutant. Conversely, the genes differentially expressed in *var2* overlap substantially with the other mutants—only 33.4% of up-regulated and 51.5% of down-regulated genes are only detected in the *var2* dataset.

Of the original 26 genes identified as over-expressed in *msl2msl3, crl*, and *ggps1*, seven (indicated with asterisks in Table 2) are also differentially expressed in *var2*. These include three cell wall-localized proteins and four potential transcription factors. At1g65500, the only gene over-expressed in *msl2msl3, crl, ggps1*, and *sca3* (indicated with a pound sign in Table 2), encodes an unknown protein. Individual comparisons between *var2* and other single mutants show the most overlap with *ggps1*, and this likely reflects the larger number of up-regulated genes found in that genotype (Figure 3). Two of the four genes identified as down-regulated in *msl2msl3, crl*, and *ggps1* are also down-regulated in the *var2* mutant. These include the cell wall-localized glycosyl hydrolyase encoded by At345940 and the oxidase encoded by At1g12010 (Table 3).

To ensure that relevant pathway genes were not excluded due to overly stringent fold-change cutoffs, this entire analysis was repeated using an absolute LFC of ±1.17 or greater and an FDR below 0.05 (Figures 3B,D). While this expanded the set of genes included in the comparisons, changing these parameters still did not result in the identification of any genes that were up-regulated or down-regulated in all five mutants. Thus, the absence of a common set of differentially expressed genes cannot be attributed solely to overly stringent LFC requirements.

### The list of differentially expressed genes common to *msl2msl3, crl*, and *ggps1* mutants does not overlap with a previously identified proposed retrograde signaling pathway

Mayer and colleagues performed a meta-analysis of microarray experiments on tissue from plants with genetically or pharmacologically altered chloroplast performance (Glaßer et al., 2014). They identified 39 genes as part of a core plastid retrograde signaling pathway that involves auxin, reactive oxygen species, the phytohormone abscisic acid, and sugar signaling. To determine if any of the mutants in our study showed a similar transcript profile, we examined the 39 genes in the proposed core retrograde signaling pathway in our lists of differentially expressed genes from *msl2msl3, crl*, and *ggps1* tissue. Of the 39 genes, 29 were differentially expressed (FDR less than 0.05) in *ggps1* white tissue, 14 in *msl2msl3* double mutants, and 13 in *crl* (Supplementary Table 1). However, only four of these genes, (At5g19120, At2g17880, At3g62950, and At2g18050), were differentially expressed in all three mutant backgrounds we analyzed, and none of the four were differentially expressed in the same direction. An aspartyl protease, a chaperone DnaJ domain protein and a thioredoxin were up-regulated in *ggps1*, but down-regulated in *crl* and *msl2msl3*, while histone H1-3 showed the opposite pattern. None of these genes has been previously identified as a participant in ion transport or plastid homeostasis. It is therefore unlikely that the 39 genes identified as part of a plastid retrograde signaling pathway are involved in the leaf architecture phenotypes seen in these mutants.

## Discussion

### Understanding the underlying source of pleiotropic phenotypes in plastid dysfunction mutants

In an effort to identify genes that may be responsible for the leaf architecture phenotypes in the *msl2msl3* double mutant, we performed RNA-seq on three mutants exhibiting similar leaf architecture defects, and then compared those results with each other and with publically available microarray data from two additional mutants. For our analysis we chose genes representing three physiologically distinct pathways to defective leaf morphology: *msl2msl3*, implicated in plastid ion homeostasis; *crl*, involved in chloroplast division; and *ggps1*, required for chlorophyll synthesis (Figure 1). We then compared the transcript profile of each mutant with the goal of identifying a core set of co-regulated genes (Figure 2 and Tables 1–3). Because of the biochemical differences underlying the secondary leaf phenotypes, any genes found to show similar gene expression patterns in all mutants would make intriguing candidates for participation in a plastid dysfunction-initiated response pathway. Given that MSL2 and MSL3 are likely to function as ion channels, this approach also could identify potential plastid ion homeostasis signaling pathways.

### Thirty differentially expressed genes are found in common between the *msl2msl3, crl*, and *ggps1* mutants

Comparison of differentially expressed genes in these mutants revealed that 26 genes were up-regulated in *msl2msl3, crl* and *ggps1*, while four were down-regulated (Figure 2). The presence of an individual gene in all three differentially expressed gene lists is evidence that it is involved in the structural leaf phenotypes observed in these mutants. If a retrograde signal from a plastid to the nucleus were responsible for changes in leaf architecture, an early response to this signal would necessarily involve the activation/deactivation of transcription factors. Indeed, 7 of the 30 genes differentially expressed in *msl2msl3, crl* and *ggps1* are predicted transcription factors (Table 2). Two Ethylene Response Factor (ERF) family members are attractive candidates for components of a retrograde plastid pathway, due to their previously documented involvement in plant stress (reviewed in Wang et al., 2013). We also identified three uncharacterized members of the plant-specific NAC domain-containing transcription factor family. This large family of over 100 genes has been implicated in many developmental and abiotic stress responses, including dehydration (Tran et al., 2004), salt tolerance (Hu et al., 2006), hypoxia (Christianson et al., 2009), secondary cell wall synthesis (Zhong et al., 2006), and separation of adjacent organs (Mallory et al., 2004). These three NAC transcription factors could be involved in response to plastid dysfunction or the developmental alterations producing the lobed leaves. Another candidate gene that could impact the expression of other proteins is the down-regulated APUM9. This conserved family of proteins has been shown to repress translation of mRNA by binding to a conserved sequence within the 3′ UTR (reviewed in Abbasi et al., 2011). Related proteins in Arabidopsis have been shown to regulate the translation of proteins required for stem cell population maintenance, such as WUSCHEL, CLAVATA-1, and ZWILLE (Francischini and Quaggio, 2009). Disrupting a similar pathway within the leaf primordia could potentially lead to architectural anomalies such as rumpled or scalloped margins.

Another intriguing group of genes identified by the analysis shown in Figure 2 are those predicted to be localized to the cell wall. Because the direction and magnitude of plant cell expansion is determined by cellulose patterning and rigidity of the cell wall (Sugimoto et al., 2000), it is possible that this group of proteins could be responsible for wavy leaves or irregular leaf margins. Two of these genes are putative hydrolases, and two are putative lipases; their upregulation could alter cell wall extensibility, leading to structural defects during leaf expansion.

### Lack of evidence for a global plastid dysfunction response pathway

As alluring as these ideas are, it would be possible to construct seemingly logical connections for almost any set of genes. GO terms associated with the genes differentially expressed in *msl2msl3, crl*, and *ggps1* were analyzed for statistically over-representation but no significant enrichment for any GO term was identified. This indicates that these genes are not likely to be working together in a previously identified, coordinated biological process. It does not eliminate the possibility that some are involved in a potential retrograde signaling pathway, but their presence on this list is not sufficient to draw conclusions. We additionally note that it would require a high amount of coordinated activity within the sample to identify significant pathways using GO term analysis with such a small number of genes.

If any of the 30 co-regulated genes we identified through RNA-seq are part of a plastid dysfunction-induced response pathway, they would be expected to show similar patterns of regulation in all mutants that display altered leaf morphology. However, comparison of our RNA-seq datasets to publically available microarray data sets from the *var2* and *sca3* mutants did not support this hypothesis (Tables 2, 3, Figure 3). The *sca3* differentially expressed gene dataset contained one of the up-regulated genes and none of the down-regulated genes. The *var2* differentially expressed gene dataset contained seven of the up-regulated genes and two of the down-regulated genes from the set of 30 we identified by RNA-seq. However, no genes were found in all five datasets, even after dropping cutoffs to LFC of 1.7, corresponding to a fold-change of approximately 2.25. This suggests that there is no single molecular pathway that is responsible for the common leaf morphology phenotypes observed in our mutants.

There are several possible explanations as to why we were unable to isolate a plastid-initiated signaling pathway. First, it is possible that a single pathway does not exist. While it is appealing to propose that the similar set of pleiotropic phenotypes in the *msl2msl3, crl1*, and *ggps1* mutants should trace back to a single pathway, different types of organelle dysfunction may lead to distinct patterns of gene regulation and signaling cascades that produce phenotypic effects that appear superficially similar. In fact, retrograde signaling pathways have already been divided into those that exert “biogenic” or developmental control, and those that exert “operational” control to optimize plastid function in response to changing environmental conditions (Pogson et al., 2008). The disruption of the isoprenoid biosynthesis pathway leading to impaired chlorophyll biosynthesis in the *ggps1* mutant would likely fall into the first category. The inability of *msl2msl3* plastids to maintain an osmotic balance across the plastid membrane is more likely to trigger operational control pathways. The specific biochemical function of CRL has yet to be determined, allowing us to speculate that a lesion in this gene could lead to plastid dysfunctions that activate both biogenic and operational retrograde signaling pathways. For example, it is possible that *crl* and *msl2msl3* share a defect in plastid division while *crl* and *ggps1* might share the presence of cells completely devoid of plastids. This framework may explain the observed overlap in differential gene expression between *crl* and *msl2msl3* and *crl* and *ggps1* as well as the lack of overlap between *msl2msl3* and *ggps1*.

The proposal that no global transcriptional response to plastid dysfunction exists is further supported by comparison of our results with the 39 plastid retrograde signaling module genes reported by Glaßer et al. (2014). None of these genes showed up-regulation or down-regulation in all three of the mutants in our analysis (Supplementary Table 1). The *ggps1* mutant was the best match, with hits on 29 of the 39 genes. This is unsurprising because the interrupted molecular pathway in *ggps1*, isoprenoid biosynthesis—and by extension, chlorophyll production—is the closest match to the chloroplast biogenesis and maintenance perturbations previously examined (inhibition of tetrapyrrole synthesis, transition of low light-adapted plants to high light, inhibition of photoassimilate export, and inhibition of redox potential). The *msl2msl3* double mutant and *crl* had 14 and 13 overlapping differentially expressed genes, respectively, but no gene was differentially expressed in the same direction in all datasets. These results are indicative of a distinct mechanism for individual types of plastid dysfunction, rather than a shared molecular pathway.

Alternatively, it is possible that a common retrograde plastid dysfunction response pathway does indeed exist, but operates in a manner that does not lend itself to discovery via transcriptome analysis. Cellular components required for the leaf morphology phenotypes could be activated through post-translational modifications, either directly or through degradation of an inhibitor. Translation of pathway components may also be regulated, with little change in the amount of mRNA present. Alternatively, the temporal regulation of these signals may make them difficult to identify, with transient spikes in mRNA followed by stable proteins. It is also possible that the secondary leaf phenotypes are a result of signaling occurring in the leaf primordia. This type of system could lead to modified leaf architecture without related transcriptional changes in mature leaves.

It is also possible that microarray analysis has insufficient resolution to identify components of this pathway. While the transcriptomic data generated here utilized RNA-seq, we relied on previously published microarray results for *var2* and *sca3* to complete our analysis. The fundamental output from these two techniques is similar (a fold-change value with an indicator of statistical confidence for each differentially regulated gene) and allows easy comparison between experiments. This approach has been used previously (Kapushesky et al., 2012; Bhargava et al., 2013; Toepel et al., 2013). A drawback of this method is that the final conclusions are only as strong as the weakest dataset. In this case, it is possible that the microarray results incorporated into our meta-analysis did not contain the necessary resolution.

## Conclusions

Here we analyzed a molecular phenotype—global mRNA sequence—in several mutant lines with superficially similar leaf and morphological defects. We hoped both to understand the basis of these phenotypes and to gain insight into the genetic pathways leading from disrupted ion homeostasis, chloroplast division, or chlorophyll biosynthesis to disrupted leaf development. We were unable to find any commonalities between the genes differentially expressed in these mutants and in other mutant transcriptomes, and conclude that either there is no common plastid-to-nucleus signaling pathway in these mutants, or it is subtle and cannot be detected through transcriptomic methods. These data show that the interaction between organelle dysfunction and resulting defects in plant leaf shape is complex and may occur through several routes, and establishes that the link between defective plastid ion homeostasis and altered leaf development shares only a few common components with other plastid dysfunction response pathways. The availability of these transcriptomic data will provide a valuable tool for the future study of organelle dysfunction, ion homeostasis, and variegation.

## RNA sequencing data

Raw data from the analyses presented here have been deposited into the NCBI SRA under the accession SRP065605.

## Author contributions

DL, MW, and EH designed and interpreted experiments; DL and MW conducted experiments and analyzed the data; DL, MW, and EH wrote and revised the manuscript.

### Conflict of interest statement

The authors declare that the research was conducted in the absence of any commercial or financial relationships that could be construed as a potential conflict of interest.

## Abbreviations

LFC: Log_2_ Fold Change
FDR: False Discovery Rate
MSL: MscS-Like
CRL: Crumpled Leaf
GGPS: Geranyl Geranyl Diphosphate Synthase
RNA-seq: RNA sequencing.
