# Supplementary material for: RNA Sequencing Analysis of the msl2msl3, crl, and ggps1 Mutants Indicates that Diverse Sources of Plastid Dysfunction Do Not Alter Leaf Morphology Through a Common Signaling Pathway
Source: Front Plant Sci. 2015 Dec 22;6:1148. doi: 10.3389/fpls.2015.01148 (PMC4686620; doi:10.3389/fpls.2015.01148)
Supplement: Supplementary file 1 [file Table1.DOCX]

**Supplementary Table 1. Log_2_ Fold Change values of the putative retrograde core response module identified by Gläßer et al. (2014) in *msl2 msl3, crl,* and *ggps1.*** Red boxes indicate a gene with a log_2_ fold change of 1.0 or higher. Blue indicates a gene with a log_2_ fold change of -1.0 or lower. Grey boxes indicate an FDR < 0.05. Bold type indicates a logFC that has a corresponding FDR < 0.05.

| **ensembl_ gene_id** | **Gene name** | **description** | ***ggps1* LFC** | ***ggps1* FDR** | ***crl* LFC** | ***crl* FDR** | ***msl2 msl3* LFC** | ***msl2 msl3* FDR** |
| --- | --- | --- | --- | --- | --- | --- | --- | --- |
| AT2G05540 |  | Glycine-rich protein family | **5.31** | 1.09e^-83^ | -2.48e^-01^ | 8.27e^-01^ | **-1.26** | 4.14e^-02^ |
| AT1G11260 | STP1 | sugar transporter 1 | **4.75** | 4.05e^-44^ | -9.65e^-01^ | 3.73e^-01^ | -7.94e^-01^ | 4.61e^-01^ |
| AT5G19120 |  | Eukaryotic aspartyl protease family protein | **3.72** | 1.25e^-29^ | **-7.48e^-01^** | 4.48e^-02^ | **-9.14**e^-01^ | 1.03e^-02^ |
| AT5G20250 | DIN10 | Raffinose synthase family protein | **5.39** | 5.57e^-29^ | -1.28 | 3.61e^-01^ | -5.43e^-01^ | 7.33e^-01^ |
| AT5G22920 |  | CHY-type/CTCHY-type/RING-type Zinc finger protein | **4.60** | 1.15e^-25^ | -9.73e^-01^ | 1.34e^-01^ | -9.85e^-01^ | 7.91e^-02^ |
| AT5G24660 | LSU2 | response to low sulfur 2 | **-4.41** | 6.29e^-24^ | -2.29e^-01^ | 9.37e^-01^ | **-1.99** | 2.76e^-02^ |
| AT2G17880 |  | Chaperone DnaJ-domain superfamily protein | **2.79** | 1.19e^-23^ | **-9.04e^-01^** | 4.25e^-02^ | **-1.22** | 2.17e^-03^ |
| AT5G21170 | AKINBETA1 | 5'-AMP-activated protein kinase beta-2 subunit protein | **3.09** | 9.77e^-23^ | -6.36e^-01^ | 3.53e^-01^ | -5.58e^-01^ | 4.05e^-01^ |
| AT1G25400 |  | unknown protein | **2.40** | 8.61e^-19^ | -1.86e^-01^ | 8.63e^-01^ | **-1.24** | 1.22e^-02^ |
| AT3G62950 | GRXC11 | Thioredoxin superfamily protein | **4.41** | 1.48e^-16^ | **-9.84e^-01^** | 1.27e^-02^ | **-2.83** | 1.75e^-14^ |
| AT1G63180 | UGE3 | UDP-D-glucose/UDP-D-galactose 4-epimerase 3 | **1.74** | 3.78e^-12^ | -3.13e^-02^ | 1.00 | -8.70e^-02^ | 9.29e^-01^ |
| AT4G17245 |  | RING/U-box superfamily protein | **1.92** | 1.99e^-10^ | 1.37e^-01^ | 8.18e^-01^ | -4.80e^-01^ | 1.94e^-01^ |
| AT3G23030 | IAA2 | indole-3-acetic acid inducible 2 | **1.94** | 4.22e^-09^ | -5.62e^-01^ | 1.62e^-01^ | 8.79e^-02^ | 9.14e^-01^ |
| AT1G49230 | ATL78 | RING/U-box superfamily protein | **-1.98** | 1.65e^-08^ | -1.74e^-01^ | 7.38e^-01^ | -4.22e^-01^ | 2.66e^-01^ |
| AT3G52740 |  | unknown protein | **-1.82** | 8.20e^-08^ | -5.38e^-01^ | 6.67e^-01^ | -9.07e^-01^ | 3.76e^-01^ |
| AT4G32280 | IAA29 | indole-3-acetic acid inducible 29 | **1.88** | 1.17e^-07^ | **-2.14** | 4.36e^-05^ | 9.56e^-01^ | 1.30e^-01^ |
| AT5G08350 |  | GRAM domain-containing protein / ABA-responsive protein-related | **2.45** | 2.44e^-07^ | -3.97e^-01^ | 4.78e^-01^ | **-9.68e^-01^** | 2.77e^-02^ |
| AT5G63190 |  | MA3 domain-containing protein | **1.01** | 8.03e^-07^ | 3.67e^-01^ | 3.58e^-01^ | 5.13e^-01^ | 1.30e^-01^ |
| AT2G18050 | HIS1-3 | histone H1-3 | **-1.15** | 1.57e^-06^ | **1.35** | 4.61e^-03^ | **2.17** | 3.91e^-07^ |
| AT5G67420 | LBD37 | LOB domain-containing protein 37 | **1.07** | 4.54e^-06^ | -3.86e^-01^ | 5.55e^-01^ | -1.55e^-01^ | 8.62e^-01^ |
| AT2G15890 | MEE14 | maternal effect embryo arrest 14 | **1.08** | 1.34e^-04^ | -3.93e^-01^ | 3.47e^-01^ | 1.73e^-02^ | 1.00 |
| AT1G52720 |  | unknown protein | **1.17** | 1.20e^-03^ | 1.12e^-01^ | 7.77e^-01^ | -3.10e^-01^ | 2.50e^-01^ |
| AT3G29030 | ATEXPA5 | expansin A5 | **-1.12** | 1.66e^-03^ | -3.14e^-02^ | 9.95e^-01^ | **-7.08e^-01^** | 5.74e^-04^ |
| AT5G64640 | PME64 | Plant invertase/pectin methylesterase inhibitor superfamily | **-6.32e^-01^** | 4.55e^-03^ | -3.02e^-01^ | 3.07e^-01^ | -6.40e^-02^ | 9.01e^-01^ |
| AT5G17860 | CAX7 | calcium exchanger 7 | **8.66e^-01^** | 6.60e^-03^ | **1.57** | 9.85e^-04^ | 5.48e^-01^ | 3.83e^-01^ |
| AT1G56510 | WRR4 | Disease resistance protein (TIR-NBS-LRR class) | **1.11** | 2.32e^-02^ | 3.42e^-01^ | 4.31e^-01^ | **-5.18** | 8.08e^-65^ |
| AT4G28270 | ATRMA2 | RING membrane-anchor 2 | **5.92e^-01^** | 2.36e^-02^ | -1.18e^-01^ | 7.98e^-01^ | **6.24e^-01^** | 1.35e^-02^ |
| AT5G51460 | ATTPPA | Haloacid dehalogenase-like hydrolase (HAD) superfamily protein | **6.84e^-01^** | 4.27e^-02^ | **-8.39e^-01^** | 4.52e^-02^ | 1.45e^-02^ | 1.00 |
| AT5G37770 | TCH2 | EF hand calcium-binding protein family | -4.53e^-01^ | 6.54e^-02^ | 1.64e^-01^ | 8.76e^-01^ | 1.32e^-01^ | 9.01e^-01^ |
| AT2G14660 |  | unknown | -8.39e^-01^ | 7.61e^-02^ | -9.06e^-02^ | 9.15e^-01^ | 1.89e^-01^ | 6.97e^-01^ |
| AT3G26740 | CCL | CCR-like | 4.00e^-01^ | 1.66e^-01^ | -1.65e^-01^ | 6.84e^-01^ | 2.65e^-01^ | 4.30e^-01^ |
| AT1G77690 | LAX3 | like AUX1 3 | -6.48e^-01^ | 1.91e^-01^ | **-7.02e^-01^** | 4.19e^-03^ | 4.46e^-01^ | 8.08e^-02^ |
| AT4G34770 |  | SAUR-like auxin-responsive protein family | -8.39e^-01^ | 3.33e^-01^ | **-1.41** | 4.26e^-05^ | 3.93e^-01^ | 3.53e^-01^ |
| AT4G04630 |  | Protein of unknown function, DUF584 | -2.83e^-01^ | 3.63e^-01^ | **-8.44e^-01^** | 1.60e^-02^ | 3.04e^-01^ | 4.73e^-01^ |
| AT3G47420 | ATPS3 | phosphate starvation-induced gene 3 | -3.41e^-01^ | 3.75e^-01^ | 5.02e^-01^ | 6.01e^-01^ | 1.16 | 9.39e^-02^ |
| AT1G66100 |  | Plant thionin | -3.05e^-01^ | 4.20e^-01^ | **8.35e^-01^** | 3.59e^-02^ | **-2.18** | 1.24e^-10^ |
| AT4G19380 | FAO4A | Long-chain fatty alcohol dehydrogenase family protein | -4.46e^-01^ | 5.52e^-01^ | 2.46e^-01^ | 5.25e^-01^ | -5.19e^-01^ | 8.82e^-02^ |
| AT4G39030 | EDS5 | MATE efflux family protein | -1.45e^-01^ | 8.77e^-01^ | **1.72** | 1.31e^-06^ | **-1.20** | 2.54e^-03^ |
| AT5G02760 |  | Protein phosphatase 2C family protein | -2.49e^-02^ | 9.93e^-01^ | **-1.91** | 9.10e^-07^ | **8.76e^-01^** | 4.44e^-02^ |
